# Supplementary material for: On/off switching of bit readout in bias-enhanced tunnel magneto-Seebeck effect
Source: Sci Rep. 2015 Mar 10;5:8945. doi: 10.1038/srep08945 (PMC4354167; doi:10.1038/srep08945)
Supplement: Supplementary Information [file srep08945-s1.pdf]

Supplementary information

On/off switching of bit readout in bias-enhanced tunnel magneto-Seebeck effect

Alexander Boehnke*[1], Marius Milnikel[2], Marvin von der Ehe[2], Christian Franz[3], Vladyslav Zbarsky[2], Michael Czerner[3], Karsten Rott[1], Andy Thomas[4], Christian Heiliger[3], Günter Reiss[1], and Markus Münzenberg[2]

1. Center for Spinelectronic Materials and Devices, Physics Department, Bielefeld University, Universitätsstrasse 25, Bielefeld, Germany

2. I. Physikalisches Institut, Georg-August-Universität Göttingen, Friedrich-Hund-Platz 1, Göttingen, Germany and Institut für Physik, Ernst-Moritz-Arndt Universität, Felix-Hausdorff-Str. 6, Greifswald, Germany

3. I. Physikalisches Institut, Justus-Liebig-Universität Gießen, Heinrich-Buff-Ring 16, Gießen, Germany

4. Thin films and Physics of Nanostructures, Physics Department, Bielefeld University, Universitätsstrasse 25, Bielefeld, Germany and Institut für Physik, Johannes Gutenberg Universität Mainz, Staudingerweg 7, Mainz, Germany

*boehnke@physik.uni-bielefeld.de

Components of the measured signal

We applied an external DC bias voltage V while simultaneously heating the top of the junction with a modulated laser. The current $I_{\mathrm{on}}(V, T_L, T_R)$ during the heating periods differs from the current $I_{\mathrm{off}}(V, T_0)$ running through the non-heated MTJ. I_{off} can be interpreted as a DC background current generated by the DC bias voltage V and depends on the resistance $R_{\mathrm{off}}(T_0)$ for the non-heated MTJ at temperature T_0. I_{on} is an AC current modulated on top of I_{off} due to the heating. It consists of a Seebeck current generated by the temperature difference $\Delta T = T_L - T_R$ and a current created by the bias voltage V dependent on the resistance $R_{\mathrm{on}}(\bar{T})$ for the mean temperature $\bar{T} = \frac{1}{2}(T_L + T_R)$ during the heating periods. The amplitude of the AC current can be expressed by:

$$\Delta I = I_{\text{on}}(V, T_L, T_R) - I_{\text{off}}(V, T_0) = I(V, \bar{T}) - I(V, T_0) + I(V, T_L, T_R) - I(V, \bar{T}) \quad \text{(i)}$$

$$= \Delta I_{\Delta \bar{T}} + \Delta I_{\Delta T}.$$

From this equation it becomes obvious that the amplitude of the AC current consists of two contributions: $\Delta I_{\Delta \bar{T}}$ results from a change in the resistance of the junction caused by the change of the mean temperature. This contribution rises linearly with applied bias voltage and vanishes at zero bias. $\Delta I_{\Delta T}$ is created by the temperature gradient and is thus related to the Seebeck effect. Eq. (1) in the main text can be deduced from Eq. (i) and describes the processes relevant for the measurements.

In the experimental setup we use a lock-in amplifier to detect the currents. Therefore we can directly measure the amplitude of ΔI. Nevertheless, Eq. (1) exhibits that the signal contains information on the Seebeck effect and on the change of the resistance. They can only be separated by their symmetry. The non-Seebeck signal behaves linearly with increasing bias when the resistance is assumed to be constant with respect to the applied voltages (Fig. 2 c). Accordingly, we can apply a linear model to separate this contribution from the overall current ΔI.

Direct and indirect determination of the Seebeck voltages

A direct measurement of the Seebeck voltage $S\Delta T$ is only possible when no external bias voltage is applied to the MTJ, but $S\Delta T$ can also be determined indirectly from the current and the resistance measurements (Eq. 1)[1,2]. Accordingly, we can compare both techniques when no bias voltage is applied to the MTJ. For zero bias voltage ($V = 0$ mV) Eq. (1) gives $S\Delta T = 1/G \cdot \Delta I = R \cdot \Delta I$. Fig. I a shows a comparison of the directly and indirectly determined $S\Delta T$. The spikes in the curve of the indirect determination occur because of slight differences in the switching fields

for the bTMS (current) and TMR effect measurements (Fig. I b & c). The measured and indirectly determined Seebeck voltages have nearly the same height. Hence, a determination of the $S\Delta T$ from the current with this method (based on Eq. (1)) leads to the correct deduction of the Seebeck coefficients and their dependence on the bias voltage.

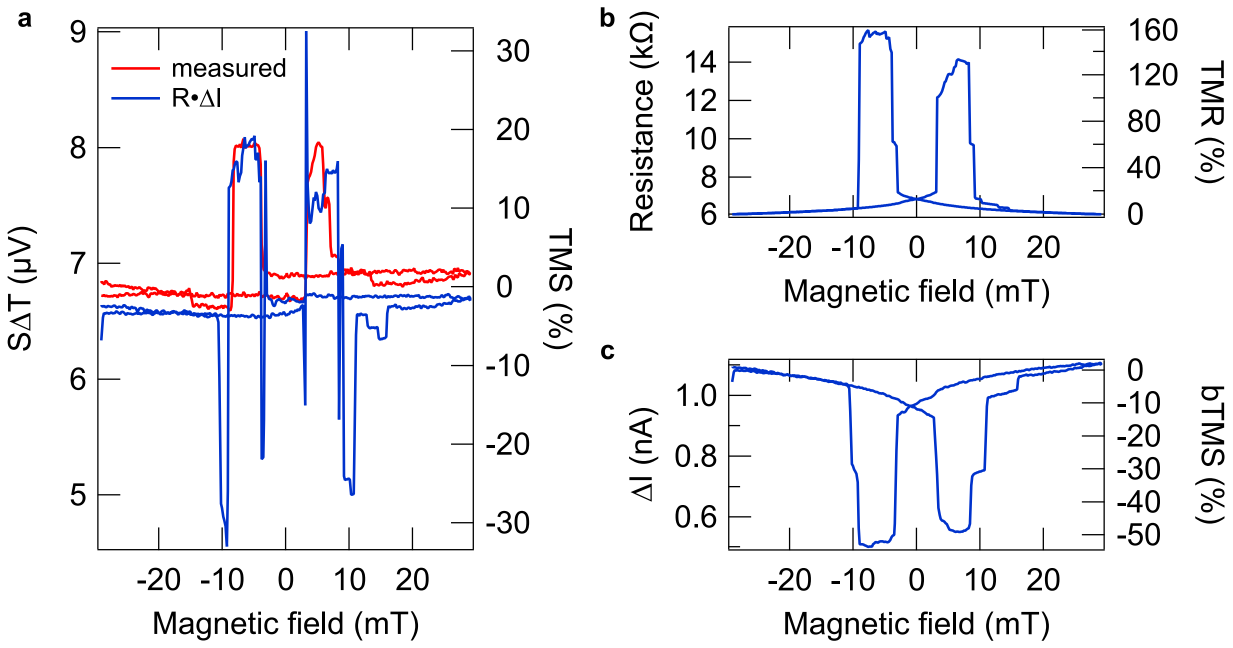

Fig I Directly and indirectly determined Seebeck voltages without bias: a The measured Seebeck voltage ($\boldsymbol{S\Delta T}$) and the $\boldsymbol{S\Delta T}$ for $\boldsymbol{V_{\text{bias}} = 0\ \text{mV}}$ determined from the current and resistance measurements show the same switching fields and the same height. The corresponding TMS ratio is given on the right. **b** Dependence of the resistance $\boldsymbol{R}$ on the applied magnetic field and corresponding TMR ratio. The measurements were performed with a bias voltage of 10 mV. **c** Dependence of the Seebeck current $\boldsymbol{\Delta I}$ on the applied magnetic field without bias voltage and corresponding bTMS ratio. Accordingly, the measured current $\boldsymbol{\Delta I = (1/R_{on}) \cdot S\Delta T}$ is proportional to the Seebeck coefficient and the inverse of the resistance ($\boldsymbol{S/R}$). Hence, the measurements of b and c can be used to determine the blue curve in a.

On/off characteristics of the current

In a DC measurement, the current through the MTJ can be written as: [3,4]

$$I_{\text{P,AP}} = G_{\text{P,AP}}(V_{\text{bias}} + S_{\text{P,AP}}\Delta T) \qquad \text{(ii)}$$

When we set $V_{\text{bias}} = -S_{\text{P}}\Delta T$ the measured current I_{P} can be set to zero. If we now reverse the magnetic state of the MTJ under a fixed bias voltage, we will find $I_{AP} = G_{AP}(S_{AP} - S_P)\Delta T$ which

is non-zero.

In our experiment we use an AC heating and measure the difference between the current when the heating is switched on and off. Accordingly, we have to rewrite Eq. (ii) to Eq. (1):

$$\Delta I = \frac{1}{R_{P,AP} - \Delta R_{P,AP}}\left(S_{P,AP}\Delta T + \frac{\Delta R_{P,AP}}{R_{P,AP}} V\right)$$

To get a zero ΔI_P we have to set the external bias voltage to $V = -S_P \Delta T \cdot (R_P/\Delta R_P)$. When the magnetic state of the MTJ is reversed and V is fixed, the current changes to

$$\Delta I_{AP} = \frac{1}{R_{AP}-\Delta R_{AP}}\left(S_{AP}\Delta T + \frac{\Delta R_{AP}}{R_{AP}} \cdot (-S_P\Delta T) \cdot \left(\frac{R_P}{\Delta R_P}\right)\right) \tag{iii}$$

$$= \frac{1}{R_{AP} - \Delta R_{AP}}\left(S_{AP} - \underbrace{\frac{\Delta R_{AP} \cdot R_P}{\Delta R_P \cdot R_{AP}}}_{\alpha} S_P\right)\Delta T.$$

As a first approximation, we can use the resistance determined by the differential conductance measurements (Fig. 2 b) and the Seebeck voltages measured without a bias voltage (Fig. I a). This gives a factor α for S_P of approximately 6.69. Inserting $S_{AP}\Delta T \approx 8$ µV and $S_P\Delta T \approx 6.8$ µV this yields a current in the AP state of the MTJ of $\Delta I_{AP} \approx 3$ nA. A comparable value for ΔI_{AP} has been measured for a bias voltage of -10 mV, where we obtain a ΔI_P of approximately zero (Fig. 2 a & b) in the P state of the MTJ. A cancelation of the TMR and TMS effects in the AP state is not seen for this MTJ. When the MTJ is switched from the P to the AP state, the changing resistances contribute a factor of $\alpha \approx 6.7$ to Eq. (iii), whereas the Seebeck voltages change by a factor of 1.2. Hence, the bracket in Eq. (iii) is zero in the P state and non-zero in the AP state.

Peltier and Thomson effects

$$\dot{Q} = \Pi \cdot I_{\mathrm{DC}}, \quad \Pi = ST. \tag{iv}$$

For the correct interpretation of our results it is essential to calculate the heat current created by the DC charge current I_{DC} driven through the MTJ by the bias voltage (Peltier effect). The amount of heat generated is directly proportional to the Peltier coefficient Π and, therefore, to the Seebeck coefficient S of the MTJ. At temperatures of $T \approx 400$ K the measured Seebeck coefficients for CoFeB/MgO MTJs are in the range of 100 μVK^{-1} to 770 μVK^{-1} [1,8]. For a minimal measured resistance of 6 kΩ and a maximal applied bias voltage of 300 mV, this yields a maximum heat current of $Q_{max} \approx 16$ µW. Thus, the heat generated by Peltier effects can be neglected, as a laser with a power of up to 150 mW is focused on top of the MTJ, creating a much larger temperature difference across the barrier than the Peltier effect.

Furthermore, a Thomson heat is generated by the temperature gradient and the current density j caused by the bias and the Seebeck voltages across the MTJ. This effect is described by the heat production rate per unit volume as

$$\dot{q} = -Kj\nabla T, K = T\frac{\mathrm{d}S}{\mathrm{d}T}, \quad \text{(v)}$$

when Joule heating and thermal conductivity are not included. K is the Thomson coefficient that is non-zero for Seebeck coefficients which depend on the temperature. For MTJs, the temperature dependence of the Seebeck coefficients has not been experimentally determined. *Ab initio* calculations[5] show that between 300 K and 400 K the Seebeck coefficients remain nearly constant for most Co-Fe compositions[4]. This yields $\mathrm{d}S/\mathrm{d}T \approx 0$ and therefore Thomson effects should vanish.

Tunnel magnetoresistance

Resistance measurements were performed with a Keithley 2400 Sourcemeter. A constant bias voltage is applied to the MTJ while the current is measured. An external magnetic field is used to

switch the relative magnetization alignment of the ferromagnetic layers between the parallel (P) and antiparallel (AP) state.

The resistance varies between R_P≈6 kΩ in the P and R_{AP}≈16 kΩ in the AP state. To determine the dependence of $R_{P,AP}$ on a wider range of V_{Bias}, measurements of R_P and R_{AP} were taken at varying V_{Bias}. The resistance is calculated from the recorded currents and the TMR ratio in dependence of the bias voltage can be obtained.

Bias enhanced tunnel magneto-Seebeck effect

We measured the bias enhanced tunnel-magneto Seebeck effect for different laser powers at a second similar sample. The data presented in Fig. II shows the measured currents ΔI for different laser powers. Fig. II a displays the dependence of the measured currents ΔI on the applied bias voltage for different laser powers. The absolute value of ΔI is always larger in the AP state of the MTJ than in the P state. At this sample, we also found a zero-crossing of the current in one magnetic state at bias voltages of approximately -10 mV and -2 mV ($\Delta I_{AP}(-10\text{ mV}) \approx 0$ nA, $\Delta I_P(-2\text{ mV}) \approx 0$ nA). This on/off characteristics leads to high (theoretically diverging) bTMS ratios at these values of the bias voltage (Fig. II b). The zero-crossing of the current and the high bTMS effect ratios originate from a compensation of the thermal current and the current created by the bias voltage. Fig. II c shows that the absolute current at a bias voltage of -10 mV increases with rising laser power. The current in the P state is much smaller than in the AP state and has an opposite sign. The increase in both states can be explained by the larger base temperature and temperature gradient that is created when the laser power is raised. The larger temperatures lead to an increased ΔI according to Eq. (1), because the Seebeck contribution $S\Delta T$ and the difference of the resistance ΔR are increased. The current in the P state was set to a value close to zero by

applying a bias voltage of -10 mV. The increase of this current exhibits that the Seebeck contribution to the overall current is rising with increasing laser power and cannot be compensated by the bias voltage contribution anymore. The current in the P state rises by a factor of 2 while the current in the AP state increases by a factor of 4.6, which leads to the observation of the highest bTMS ratio at a laser power of 150 mW and a bias voltage of -10 mV.

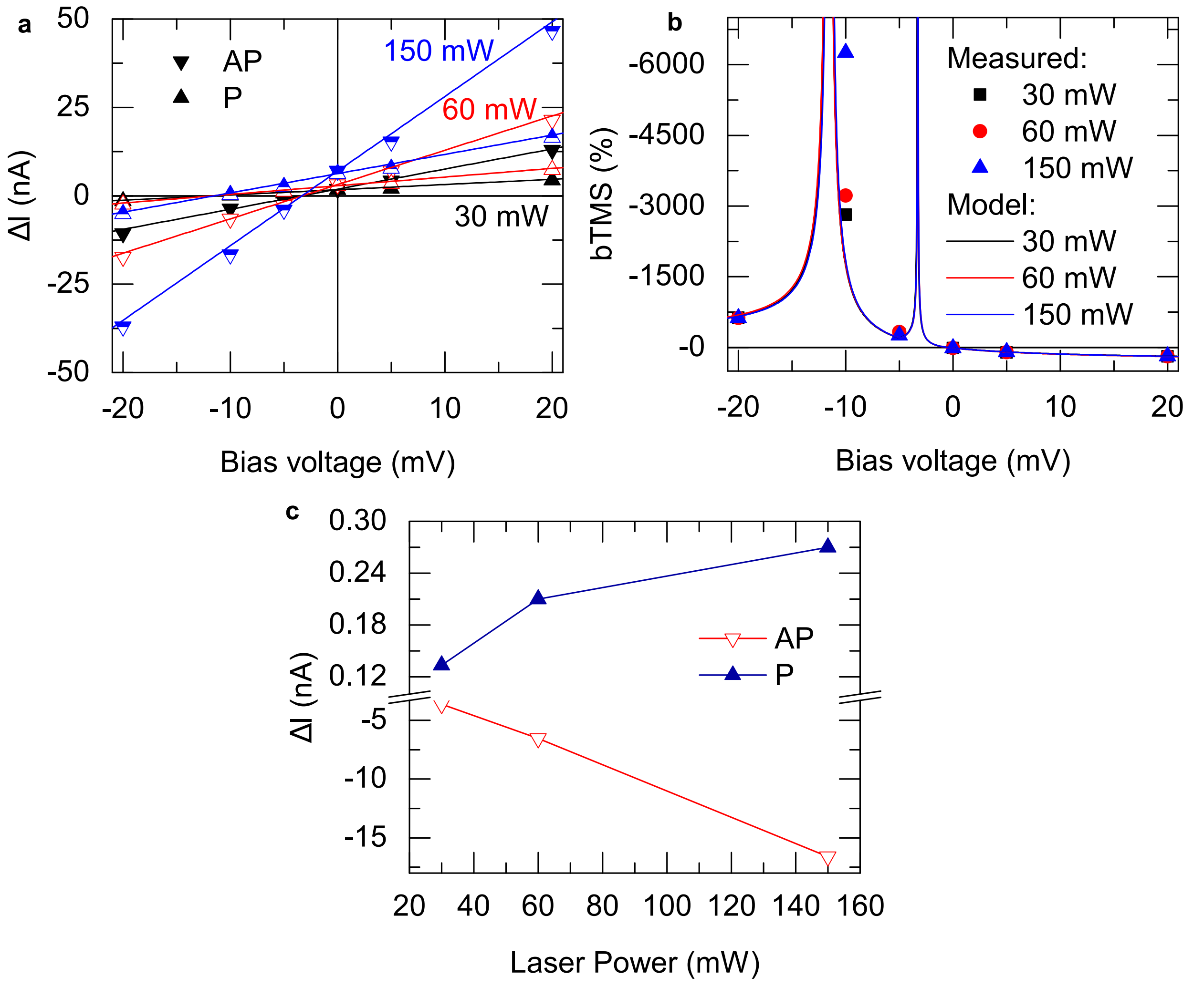

Fig. II Laser power dependence of the bias enhanced TMS effect: a Dependence of the current ΔI on the bias voltage for different laser powers. A zero-crossing of the current for one magnetic state can be observed at approximately -10 mV and -2 mV. The absolute current rises with increasing laser power. **b** bTMS ratio determined from the measurements in a. The highest effect of more than -6000% is observed for a laser power of 150 mW. **c** Current measurements at an applied bias voltage of -10 mV. At this value of the bias voltage the current in the P state is close to zero, whereas, the current in the AP state is two orders of magnitude larger. The increase of the current in the P state shows that the Seebeck and the voltage contribution compensate better for smaller laser powers.

Contributions of bias voltage and Seebeck voltage signal

In the experiment we found a zero current singal in the P state at a bias voltage of -10 mV ($\mathit{\Delta} I_\mathrm{P}(-10\ \mathrm{mV}) \approx 0\ \mathrm{nA}$). We can calculate the corresponding Seebeck voltage $S_\mathrm{P}\mathit{\Delta} T$ that is compensated by the bias votlage of -10 mV using Eq. (1). Further we need the measured conductances for the MTJ in the P state $G_\mathrm{on} \approx 194.97\ \mu\mathrm{S}$ and $G_\mathrm{off} \approx 194.70\ \mu\mathrm{S}$ when the laser is switched on or off.

$$\frac{\mathit{\Delta} I}{G_\mathrm{on}} - \frac{G_\mathrm{on} - G_\mathrm{off}}{G_\mathrm{on}} V = S \mathit{\Delta} T$$

$$S_\mathrm{P}\mathit{\Delta} T \approx \frac{0.27\ \mu\mathrm{V}}{194.97\ \mu\mathrm{V}} \cdot (-10\ \mathrm{mV}) \approx 13.85\ \mu\mathrm{V}.$$

The same calculation can be done for the AP state of the MTJ where a bias voltage of -2 mV is needed to componstate the current signal $\mathit{\Delta} I_\mathrm{AP}(-2\ \mathrm{mV}) \approx 0\ \mathrm{nA}$. The conductances for the AP sate are $G_\mathrm{on} \approx 81.02\ \mu\mathrm{S}$ and $G_\mathrm{off} \approx 80.18\ \mu\mathrm{S}$.

$$S_\mathrm{AP}\mathit{\Delta} T \approx \frac{0.84\ \mu\mathrm{S}}{81.02\ \mu\mathrm{S}} \cdot (-2\ \mathrm{mV}) \approx 20.8\ \mu\mathrm{V}.$$

Because of the small factors $(G_\mathrm{on} - G_\mathrm{off})/G_\mathrm{on}$ relatively high voltages in the millivolt regime are needed to compensate the contirbution of the Seebeck voltages in the microvolt range to the measured currents $\mathit{\Delta} I$. Seebeck voltages of some microvolts are measured at the investigated junctions when no bias voltage is applied (Fig. I a).

Bibliography

1. Boehnke, A. *et al.* Time-resolved measurement of the tunnel magneto-Seebeck effect in a single magnetic tunnel junction. *Rev. Sci. Instrum.* **84,** 063905 (2013).

2. Liebing, N. *et al.* Tunneling magneto thermocurrent in CoFeB/MgO/CoFeB based magnetic tunnel junctions. *Appl. Phys. Lett.* **102,** 242413 (2013).

3. Johnson, M. Spin caloritronics and the thermomagnetoelectric system. *Solid State Commun.* **150,** 543–547 (2010).

4. Johnson, M. & Silsbee, R. H. Thermodynamic analysis of interfacial transport and of the thermomagnetoelectric system. *Phys. Rev. B* **35,** 4959–4972 (1987).

5. Heiliger, C., Franz, C. & Czerner, M. Ab initio studies of the tunneling magneto-Seebeck effect: Influence of magnetic material. *Phys. Rev. B* **87,** 224412 (2013).
